# Children with Reading Disability Show Brain Differences in Effective Connectivity for Visual, but Not Auditory Word Comprehension

**DOI:** 10.1371/journal.pone.0013492

**Published:** 2010-10-25

**Authors:** Li Liu, Amit Vira, Emma Friedman, Jennifer Minas, Donald Bolger, Tali Bitan, James Booth

**Affiliations:** 1 State Key Laboratory of Cognitive Neuroscience and Learning, Beijing Normal University, Beijing, People's Republic of China; 2 Department of Communication Sciences and Disorders, Northwestern University, Evanston, Illinois, United States of America; 3 Department of Human Development, Maryland University, College Park, Maryland, United States of America; 4 Department of Communication Disorders, Haifa University, Haifa, Israel; Indiana University, United States of America

## Abstract

**Background:**

Previous literature suggests that those with reading disability (RD) have more pronounced deficits during semantic processing in reading as compared to listening comprehension. This discrepancy has been supported by recent neuroimaging studies showing abnormal activity in RD during semantic processing in the visual but not in the auditory modality. Whether effective connectivity between brain regions in RD could also show this pattern of discrepancy has not been investigated.

**Methodology/Principal Findings:**

Children (8- to 14-year-olds) were given a semantic task in the visual and auditory modality that required an association judgment as to whether two sequentially presented words were associated. Effective connectivity was investigated using Dynamic Causal Modeling (DCM) on functional magnetic resonance imaging (fMRI) data. Bayesian Model Selection (BMS) was used separately for each modality to find a winning family of DCM models separately for typically developing (TD) and RD children. BMS yielded the same winning family with modulatory effects on bottom-up connections from the input regions to middle temporal gyrus (MTG) and inferior frontal gyrus(IFG) with inconclusive evidence regarding top-down modulations. Bayesian Model Averaging (BMA) was thus conducted across models in this winning family and compared across groups. The bottom-up effect from the fusiform gyrus (FG) to MTG rather than the top-down effect from IFG to MTG was stronger in TD compared to RD for the visual modality. The stronger bottom-up influence in TD was only evident for related word pairs but not for unrelated pairs. No group differences were noted in the auditory modality.

**Conclusions/Significance:**

This study revealed a modality-specific deficit for children with RD in bottom-up effective connectivity from orthographic to semantic processing regions. There were no group differences in connectivity from frontal regions, suggesting that the core deficit in RD is not in top-down modulation.

## Introduction

Reading disability (RD) refers to a significant reading difficulty that cannot be accounted for by deficits in general intelligence or education. Previous literature suggests that those with reading disability have more pronounced deficits during semantic processing in reading as compared to listening comprehension [1]–[4]. This discrepancy between reading and listening comprehension has also been supported by recent neuroimaging studies. Studies investigating semantic processing tasks in the visual modality have reported alterations of activation in reading disability in posterior regions such as middle temporal and inferior parietal cortex [5]–[8] and frontal regions such as inferior frontal gyrus [7], [9]. Unlike studies in the visual modality, studies on semantic processing in the auditory modality generally have shown modest group differences or failed to show any group differences between children with reading disability and typically developing children [9]–[11].

Accumulating evidence from connectivity studies suggests that those with reading disability have deficits in the interaction among brain regions. Two studies have used functional connectivity analyses to examine non-directional correlations between brain regions. Horwitz et al. (1998) reported that adults with reading disabilities did not show a correlation of left angular gyrus with left inferior frontal gyrus or with left fusiform gyrus as controls did during single word naming [12]. Similarly, Pugh et al. (2000) reported that adults with reading disabilities did not show a correlation of left angular gyrus with lingual gyrus as controls did during a nonword rhyming judgment task and a semantic category judgment task [13]. Some studies have used effective connectivity methods to examine directionally specific influences between brain regions. Cao et al. (2008) reported that effective connectivity from left fusiform gyrus to left inferior parietal lobule, but not from left inferior frontal gyrus to left inferior parietal lobule, was stronger in controls than in children with reading difficulties during word rhyming [14], suggesting a bottom-up deficit in reading disability. Quaglino et al. (2008) reported disrupted effective connectivity from inferior parietal lobule to inferior frontal gyrus in a pseudoword reading task in a dyslexic group compared to chronological age- and reading level-matched groups, also suggesting a bottom-up deficit from posterior regions to frontal cortex in reading disability [15].

In summary, these connectivity studies consistently reported disrupted connectivity in temporal-parietal regions (e.g. angular gyrus, inferior parietal lobule) with frontal cortex (e.g. inferior frontal gyrus) or with visual association cortex (e.g. fusiform gyrus, lingual gyrus) In addition, the two effective connectivity studies indicated that bottom-up rather than top-down connectivity is affected in reading disability. However, these studies have mainly used phonological tasks; no previous studies have used effective connectivity methods to examine directionally specific effects during semantic processing.

The goal of this study was first to replicate the discrepancy between reading and listening word comprehension in reading disability. This was achieved by examining effective connectivity during a semantic relatedness judgment task in the visual and auditory modality. This study is a re-analysis of a subset of the data from our previous paper [9] but uses Dynamic Causal Modeling that allows one to test directionally specific influences between brain regions [16], [17]. This study focused on four regions of interest including two implicated in processing modality specific input – the superior temporal gyrus involved in phonological processing [18] and the fusiform gyrus implicated in orthographic processing [19]. We examined the bottom-up influence of these regions on an area extensively implicated in semantic processing – the middle temporal gyrus [9], [20], [21] – as well as the top-down influence from inferior frontal gyrus to this semantic processing region. We expected reduced effective connectivity in reading disability only in the visual modality, but not in the auditory modality, suggesting that the disruptive access to semantic representations is modality specific.

Secondly, we wished to examine whether there are disrupted top-down influences from inferior frontal gyrus or bottom-up influences from fusiform gyrus in reading disability. Previous effective connectivity studies on phonological processing have indicated bottom-up rather than top-down connectivity deficits in reading disability [14], [15]. In addition, previous studies also suggest that differences in the inferior frontal gyrus may not represent the critical deficit in reading disability [22] and in fact this region may be used as a compensatory mechanism [6]. Based on the above, for the visual modality, we expected that there may be minimal differences between those with and without reading disability in top-down influence from inferior frontal gyrus to middle temporal gyrus, but significant differences in bottom-up influence from fusiform gyrus to middle temporal gyrus.

## Methods

### Participants

This research was approved by the Institutional Review Board at Northwestern University and Evanston Northwestern Healthcare Research. Written consent was obtained from each participant. Twelve children with reading disabilities (RD) (M age = 10.6, range = 8.08–14.09; 10 males) and 12 age-matched typically developing (TD) children (M age = 10.6, range = 8.09–14.11; 9 males) participated in this study. Parents of children also gave written informed consent for participation of their children. In addition, they were given an informal interview to insure that the children met the following inclusionary criteria: (1) native English speakers, (2) right-handedness, (3) normal hearing and normal or corrected-to-normal vision, (4) free of neurological disease or psychiatric disorders, (5) not taking medication affecting the central nervous system, and (6) no Attention Deficit Hyperactivity Disorder (ADHD). TD children had no history of reading or oral-language deficits, and the RD children had a diagnosis of learning disability by a clinical psychologist.

### Standardized testing

Mental ability was measured with the Wechsler Abbreviated Scale of Intelligence (WASI) [23] with two verbal subtests (Vocabulary, Similarities) and two performance subtests (Block Design, Matrix Reasoning). Spelling was measured by the Wide Range Achievement Test (WRAT) [24]. Word (Word Identification) and nonword (Word Attack) reading accuracy was measured with the Woodcock Johnson III Tests of Achievement (WJ-III) [25]. Word (Sight Word Efficiency) and nonword (Phonemic Decoding Efficiency) reading speed was measured by the Tests of Word Reading Efficiency (TOWRE) [26]. Phonological skills (including phonological awareness, phonological memory, and rapid naming) were measured with the Comprehensive Test of Phonological Processing (CTOPP) [27].

RD children met the following inclusionary criteria: (1) Performance or Verbal IQ above 85 - 10 of 12 children were higher than 90 on Performance IQ and 8 children were higher than 90 on Verbal IQ, (2) lower than 85 on at least one of the four standardized reading measures (Word Identification, Word Attack, Sight Word Efficiency, Phonemic Decoding Efficiency), and (3) average of the four standard reading measures below 95. The TD children met the following criteria: (1) difference of age with matched children with RD less than four months, (2) Performance IQ or Verbal IQ above 90, and (3) average of the four standardized reading measures above 95.

Table 1 presents the means and standard deviations of scaled scores of the standardized tests in the TD and RD groups. The TD and RD groups were matched on Performance IQ (F(1,23) = 2.843, p = .106), but not Verbal IQ (F(1,23) = 5.472, p = .029). There were significant differences between groups on all of achievement measures (F(1,23) = 50.185, p = .000 for Spelling; F(1,23) = 46.489, p = .000 for Word Identification; F(1,23) = 32.105, p = .000 for Word Attack; F(1,23) = 62.564, p = .000 for Sight Word Efficiency; F(1,23) = 30.229, p = .000 for Phonemic Decoding Efficiency). In order to determine if there was a larger reading achievement than verbal ability discrepancy, we calculated a 2 group (TD, RD)×2 test (average of the four standardized reading measures, Verbal IQ) ANOVA, and found a significant interaction between group and test (F(1, 22) = 9.683, p = .005). Follow-up tests showed that the group difference was large for the average of the four standard reading measures (F(1,23) = 76.336, p = .000), but small for Verbal IQ (F(1,23) = 5.472, p = .029), suggesting larger reading deficits than general verbal language deficits. There were also significant differences between groups on all of the measures for phonological skills (t(22) = 4.310, p = 0.000 for Phonological Awareness; t(22) = 3.281, p = 0.003 for Phonological Memory; t(22) = 3.594, p = 0.002 for Rapid Naming).

**Table 1:** Means (and standard deviations) for standardized test performance for typically developing (TD) and reading disability (RD) groups.

Test	TD	RD
**WASI**		
Verbal (VIQ)	106(11)	95(13)
Performance (PIQ)	107(12)	99(11)
**WRAT**		
Spelling	110(11)	82(8)
**WJ-III**		
Word reading accuracy (Word ID)	108(9)	86(6)
Nonword reading accuracy (Word Attack)	106(10)	84(9)
**TOWRE**		
Word reading speed (SWE)	106(8)	84(5)
Nonword reading speed (PDE)	103(12)	79(10)
**CTOPP**		
Phonological Awareness (PA)	105(11)	86(11)
Phonological Memory (PM)	102(13)	87(10)
Rapid naming(RN)	101(12)	84(10)

Note: WASI = Wechsler Abbreviated Intelligence Scale; VIQ = Verbal Intelligence Quotient; PIQ = Performance Intelligence Quotient; WRAT = Wide Range Achievement Test; WJ-III = Woodcock Johnson III Tests of Achievement; Word ID = Word Identification; TOWRE = Test of Word Reading Efficiency; SWE = Sight Word Efficiency; PDE = Phonemic Decoding Efficiency. CTOPP = Comprehensive Test of Phonological Processing. Standard scores are presented (M = 100, SD = 15).

### Functional activation tasks

Two word association judgment tasks, one in visual modality and the other in auditory modality, were given to all the subjects. For both modalities, two words were presented sequentially and then a red fixation-cross appeared on the screen after the second word, indicating the need to make a response during the subsequent 2,600 ms interval. In the visual modality, the duration of each word was 800 ms followed by a 200 ms blank interval. In the auditory modality, the duration of each word was between 500 and 800 ms followed by a brief period of silence, with the second word beginning 1000 ms after the onset of the first. A fixation-cross appeared throughout the trial in the auditory modality while the two words were presented sequentially. For both modalities, forty-eight word pairs were semantically related according to their free association values for the auditory (mean = .45, SD = .21, range = .85–.12) and visual modalities (mean = 0.45, SD = .19, range = .77–.14) [28]. Half of the related pairs were high association and half of them were low association according to their free association values [29]. Twenty-four word pairs were semantically unrelated with zero association values. Larger number of trials in the related condition may result in a better estimation of the brain response at the individual subject level for this condition as compared to the unrelated condition. This may have made it more likely to find group differences (TD versus RD) in this condition. In addition, it is possible that this asymmetry could have caused a response bias toward yes (related) responses; however, we did not find a response bias when examining the behavioral data. The participants were instructed to quickly and accurately press the yes button with their right hand to the related pairs and the no button to the unrelated pairs. See [30], [31] for additional details on material characteristics.

There were also two types of control trials for both modalities, one we called ‘perceptual’, and the other we called ‘baseline’. The auditory ‘perceptual’ control involved tone matching judgments (48 trials) and the visual ‘perceptual’ control involved false font matching judgments (48 trials). For both modalities, participants determined whether the pair of stimuli were identical or not by pressing a yes or no button. Both modalities also had a ‘baseline’ control with 60 trials that required a button press when a black fixation-cross turned red. See [30], [31] for additional details of the control tasks. The order of lexical and control trials was optimized for event-related design using OptSeq (http://surfer.nmr.mgh.harvard.edu/optseq) [32].

### Data collection

All images were acquired using a 1.5 T GE scanner. Each participant performed four functional runs, two in the visual modality and the other two in the auditory modality. Half of the participants completed the task in the visual modality first and half of them completed the auditory modality first. For the functional imaging, a susceptibility weighted single-shot EPI (echo planar imaging) method was used. Functional images were interleaved from bottom to top in a whole brain EPI acquisition. The following scan parameters were used: TE = 35 ms, flip angle = 90°, matrix size = 64×64, field of view = 24 cm, slice thickness = 5 mm, number of slices = 24 and TR = 2000 ms. The first functional run had 203 image volumes and the second had 198 image volumes. The first run took 6.7 min and the second 6.6 min. For the structural imaging, a high resolution, T1 weighted 3D image was acquired (SPGR, TR = 21 ms, TE = 8 ms, flip angle = 20°, matrix size = 256×256, field of view = 22 cm, slice thickness = 1 mm, number of slices = 124). The orientation of the 3D image was identical to the functional slices.

### Imaging data analysis

Data analysis was performed using SPM5 (Statistical Parametric Mapping) (http://www.fil.ion.ucl.ac.uk/spm). The functional images were corrected for differences in slice-acquisition time to the middle volume and were realigned to the first volume in the scanning session using affine transformations. No participant had more than 4.0 mm of movement within run in any plane. Coregistered images were normalized to the MNI (Montreal Neurological Institute) average template with a resampled voxel size of 2*2*2 and then smoothed with Gaussian filter of FWHM (full width half max) = 10 mm.

The general linear model was used to estimate condition effects for each subject using an event-related analysis procedure. Four conditions “related”, “unrelated”, “perceptual”, and “baseline” were modeled using a canonical HRF (hemodynamic response function). For each subject, one contrast of interest was computed: lexical (related+unrelated) vs. baseline. Parameter estimates from contrasts of the canonical HRF in single subject models were entered into random-effects analysis. One-sample t tests were used to test if a contrast was significant, separately for each group and each modality. Two-sample t tests were used to test if a contrast was significantly different between groups for each modality. Because this study is a re-analysis of a subset of published data [9] that examined differences between TD and RD in signal intensity and also because the focus of this study is on group differences, only group differences are reported (p<0.001 uncorrected, >20 voxels).

### DCM analysis: Regions of interest (ROI) specification

All DCM models in the current paper involved a three-region neural network in the left hemisphere. Left-hemisphere regions were chosen because only left-hemisphere brain regions were reported to show significant correlations with semantic association strength in the conventional analysis (See [9]). The three regions in the visual modality were: middle temporal gyrus (MTG), the anterior part of inferior frontal gyrus (IFG) and fusiform gyrus (FG). MTG and IFG were included based on the model of semantic processing proposed by Lau and colleagues (2008) [21]. In their model, they suggested that left middle temporal gyrus is involved in representing semantic information [33] and that the anterior part of left inferior frontal gyrus is involved in controlled retrieval of semantic representations. The model of Lau and colleagues (2008) also included left inferior parietal lobule, left anterior temporal cortex and the posterior part of left inferior frontal gyrus. We did not include left inferior parietal lobule in our model because this area failed to show significantly greater activation in the lexical judgment compared to baseline. We did not include left anterior temporal cortex in our model because this area seems to be more involved in sentence-level semantic processing [34] while single words were used as stimuli in the current study. In addition, we did not include left posterior inferior frontal gyrus in our model because this region has been suggested to be involved in general lexical selection rather than a region specialized for semantic processing [21]. Moreover, many studies have suggested a functional separation of the posterior dorsal versus anterior ventral aspects of left inferior frontal gyrus with the latter being critical for semantic processing [35]–[37]. FG was also included in the DCM models in the visual modality because it is thought to be associated with processing of orthography [19]. The above regions except FG were also included as regions of interests in the auditory modality. Superior temporal gyrus (STG), instead of FG, was included in the auditory modality because it is thought to be associated with processing of phonology [18].

In order to avoid biases in the identification of the ROIs towards TD group or RD group, the ROIs for the effective connectivity analysis were chosen based on activation across groups. All the regions were chosen based on the contrast of lexical (related+unrelated) vs. baseline. The group maxima (x, y, z) for the ROIs in the visual modality were MTG (-48 -51-3), IFG (-36 27 -3), and FG (-60 -12 3). The group maxima for auditory modality were MTG (-57 -48 6), IFG (-33 27 -6), and STG (-39 -69 -18).

The ROIs were specified for each individual for each modality. All ROIs were 6 mm radius spheres centered on the most significant voxel in the individuals' activation map close to the group maximum. The selection of the individual ROIs was constrained by the intersection of two masks: (1) anatomical mask of the relevant region (i.e. inferior frontal gyrus for IFG, middle temporal gyrus for MTG, fusiform gyrus for FG, superior temporal gyrus for STG); (2) spherical mask of 10 mm radius centered on the group maximum of the relevant region. The following Brodmann areas (BA) were represented across individuals for each ROI: IFG (BA 45, 47), MTG (BA 21), FG (BA 37, 19), and STG (BA 22).

### DCM analysis: Effective connectivity analysis

Effective connectivity was examined using the Dynamic Causal Modeling (DCM) tool [17] in SPM8. DCM is a nonlinear systems identification procedure that uses Bayesian estimation to make inferences about effective connectivity between neural systems and how it is affected by experimental conditions. In DCM, three sets of parameters are estimated: the direct input of stimuli on regional activity; the intrinsic connections between regions in the absence of modulating experimental effects; and the changes in the intrinsic connectivity between regions induced by the experimental design (modulatory effects) [38].

Our analysis adopted a three-stage procedure. The first stage was a comparison among alternative families of DCM using Bayesian Model Selection (BMS) and model space partitioning [39]–[41]. The second stage was Bayesian Model Averaging (BMA) analysis within the winning family [39], [41]. The third stage was parameter level analysis by entering the subject-wise BMA estimates (posterior means of parameter densities) into ANOVAs in order to determine differences between groups, between directions of influence, between modulatory effects of different lexical conditions and their interactions.

The entire model space with modulatory effects of 2 lexical conditions (related, unrelated) on 6 connections resulted in 128 models for each subject in each modality. To reduce model space, we assumed that related and unrelated conditions modulated the same connections, which reduced the model space to 64 models for each subject in each modality. Because we are mainly interested in bottom-up connections from the input region (FG for the visual modality, STG for the auditory modality) to MTG and IFG and top-down connections from IFG to MTG and the input region (FG for the visual modality, STG for the auditory modality), we divided model space into families of DCM models based on modulatory effects on these critical connections. First, we compared 4 families with or without modulatory effects on bottom-up connections. In the visual modality, the 64 models were divided into 4 families: Family 1 with modulatory effects of related and unrelated conditions on the connection from FG to MTG, but not on the connection from FG to IFG; family 2 with modulatory effects on the connection from FG to IFG, but not on the connection from FG to MTG; family 3 with modulatory effects on both connections; family 4 without modulatory effects on either connection. Similar BMS analysis was done for the auditory modality except that the input region was STG.

In the second stage we partitioned the 16 models in the winning family into 4 families based on the modulations on top-down connections: Family A with modulatory effects of related and unrelated conditions on the connection from IFG to MTG, but not on the connection from IFG to FG; family B with modulatory effects on the connection from IFG to FG, but not on the connection from IFG to MTG; family C with modulatory effects on both connections; family D without modulatory effects on either connection. Similar BMS analysis was done for the auditory modality except that the input region was STG (see Table 2 and 3 for model space in the visual and auditory modality separately).

**Table 2:** Model space in the visual modality.

	Models in family 1																Models in family 2															
	1	2	3	4	5	6	7	8	9	10	11	12	13	14	15	16	17	18	19	20	21	22	23	24	25	26	27	28	29	30	31	32
**FG→MTG**	*	*	*	*	*	*	*	*	*	*	*	*	*	*	*	*																
**FG→IFG**																	*	*	*	*	*	*	*	*	*	*	*	*	*	*	*	*
**IFG→MTG**	*	*	*	*					*	*	*	*					*	*	*	*					*	*	*	*				
**IFG→FG**					*	*	*	*	*	*	*	*									*	*	*	*	*	*	*	*				
**MTG→FG**	*		*		*		*		*		*		*		*		*		*		*		*		*		*		*		*	
**MTG→IFG**		*	*			*	*			*	*			*	*			*	*			*	*			*	*			*	*	

	Models in family 3																Models in family 4															
	Family A				Family B				Family C				Family D																			
	33	34	35	36	37	38	39	40	41	42	43	44	45	46	47	48	49	50	51	52	53	54	55	56	57	58	59	60	61	62	63	64
**FG→MTG**	*	*	*	*	*	*	*	*	*	*	*	*	*	*	*	*																
**FG→IFG**	*	*	*	*	*	*	*	*	*	*	*	*	*	*	*	*																
**IFG→MTG**	*	*	*	*					*	*	*	*					*	*	*	*					*	*	*	*				
**IFG→FG**					*	*	*	*	*	*	*	*									*	*	*	*	*	*	*	*				
**MTG→FG**	*		*		*		*		*		*		*		*		*		*		*		*		*		*		*		*	
**MTG→IFG**		*	*			*	*			*	*			*	*			*	*			*	*			*	*			*	*	

**Table 3:** Model space in the auditory modality.

	Models in family 1																Models in family 2															
	1	2	3	4	5	6	7	8	9	10	11	12	13	14	15	16	17	18	19	20	21	22	23	24	25	26	27	28	29	30	31	32
**STG→MTG**	*	*	*	*	*	*	*	*	*	*	*	*	*	*	*	*																
**STG→IFG**																	*	*	*	*	*	*	*	*	*	*	*	*	*	*	*	*
**IFG→MTG**	*	*	*	*					*	*	*	*					*	*	*	*					*	*	*	*				
**IFG→STG**					*	*	*	*	*	*	*	*									*	*	*	*	*	*	*	*				
**MTG→STG**	*		*		*		*		*		*		*		*		*		*		*		*		*		*		*		*	
**MTG→IFG**		*	*			*	*			*	*			*	*			*	*			*	*			*	*			*	*	

	Models in family 3																Models in family 4															
	Family A				Family B				Family C				Family D																			
	33	34	35	36	37	38	39	40	41	42	43	44	45	46	47	48	49	50	51	52	53	54	55	56	57	58	59	60	61	62	63	64
**STG→MTG**	*	*	*	*	*	*	*	*	*	*	*	*	*	*	*	*																
**STG→IFG**	*	*	*	*	*	*	*	*	*	*	*	*	*	*	*	*																
**IFG→MTG**	*	*	*	*					*	*	*	*					*	*	*	*					*	*	*	*				
**IFG→STG**					*	*	*	*	*	*	*	*									*	*	*	*	*	*	*	*				
**MTG→STG**	*		*		*		*		*		*		*		*		*		*		*		*		*		*		*		*	
**MTG→IFG**		*	*			*	*			*	*			*	*			*	*			*	*			*	*			*	*	

For all the above models, direct input (which includes related, unrelated and perceptual conditions) was specified on FG in the visual modality, whereas in the auditory modality the direct input was specified on STG; intrinsic connections were fully and reciprocally connected between the three ROIs in the visual modality (FG, MTG & IFG) and between the three ROIs in the auditory modality (STG, MTG & IFG).

For families that did not show significant differences in model space partitioning, random effects Bayesian model averaging (BMA) was conducted across all models in these families.

The final step in the analysis was done on parameter estimates of the averaged model resulting from the BMA, using a random effects frequentist approach. We conducted a series of ANOVAs to examine differences between the TD and RD groups in the modulatory effects of different lexical conditions (related, unrelated) across different coupled regions. We report only main effects or interactions involving group in these larger models (p<.05) because this is the focus of our study. Significant interactions in these larger models were broken down into more specific analyses and significant effects (p<.05) are noted in the data.

## Results

### Behavioral results

Table 4 presents behavioral data on the word judgment tasks. We calculated a 2 group (TD, RD)×2 modality (visual, auditory)×2 condition (related, unrelated) ANOVA on reaction times. This analysis showed that the TD group was significantly faster than the RD group (F(1,22) = 4.722, p = .041) and that related pairs were significantly faster than unrelated pairs (F(1,22) = 54.705, p = .000). There were no other significant main effects or interactions. We calculated the same ANOVA on accuracy. This analysis revealed that the TD group had higher accuracy than the RD group (F(1,22) = 4.722, p = .041). In addition, this analysis revealed a significant interaction of group by modality (F(1,22) = 9.980, p = .005). Follow-up tests showed that accuracy differences between groups were significant in the visual modality (F(1,22) = 22.436, p = .000) but not in the auditory modality (F(1,22) = 2.387, p = .137). There were no other significant main effects or interactions.

**Table 4:** Mean accuracy and reaction time (and standard deviations) for related and unrelated conditions in the visual and auditory semantic task for typically developing (TD) and reading disability (RD) groups.

	Accuracy (%)				Reaction time (ms)			
	Related		Unrelated		Related		Unrelated	
	Visual	Auditory	Visual	Auditory	Visual	Auditory	Visual	Auditory
TD	93(9)	89(11)	91(11)	83(16)	1278(362)	1353(309)	1541(384)	1484(306)
RD	74(13)	80(10)	70(25)	78(20)	1504(284)	1625(271)	1731(247)	1777(245)

### fMRI signal intensity results

The current paper is a re-analysis of a subset of data that examined differences between TD and RD children in signal intensity [9]. Twenty-one subjects (eleven subjects for the RD group and ten subjects for the TD group) are overlapping between the current study and the previous study of Booth (2007). Because many of the same subjects were used in the previous analyses, the results of the present analyses of signal intensity are similar. For the lexical (related+unrelated) versus baseline contrast in the visual modality, the TD group showed no areas of greater activation than the RD group and the RD group showed greater activation than the TD group in right medial frontal gyrus (BA 9; voxels = 43; x = 15, y = 27, z = 36; Z = 4.21), right superior frontal gyrus (BA 9; voxels = 33; x = 33, y = 33, z = 33; Z = 3.84) and right lingual gyrus (BA 18; voxels = 25; x = 12, y = −87, z = −12; Z = 3.44). The TD group showed no areas of greater activation than the RD group in the auditory modality either, but the RD group showed greater activation than the TD group in right postcentral gyrus (BA 2; voxels = 48; x = 57, y = −24, z = 45; Z = 4.58).

### Bayesian Model Selection (BMS) results

Table 5 shows the posterior family exceedance probabilities from a random effects BMS analysis in the visual modality. During the first step of BMS analysis with model space partitioning, family 3 with modulatory effects of related and unrelated conditions on both bottom-up connections (from FG to MTG and to IFG) showed the highest evidence out of the 4 families for both TD (Family 3, exceedance probabilities 0.88) and RD (Family 3, exceedance probabilities 0.76). At the next step of BMS analysis, the 16 models included in family 3 were partitioned into 4 separate families (family A, B, C, D) with different modulatory effects on top-down connections. However, there was no clear evidence in favor of any family for either TD or RD.

**Table 5:** Posterior family exceedance probabilities for the typically developing (TD) and reading disability (RD) groups in the visual modality.

		TD	RD
First step BMS	Family 1	0.09	0.07
	Family 2	0.02	0.13
	**Family 3**	**0.88**	**0.76**
	Family 4	0.01	0.04
Second step BMS	Family A	0.28	0.39
	Family B	0.27	0.34
	Family C	0.24	0.13
	Family D	0.21	0.14

Note: First step BMS was to test families with different *bottom-up* modulatory effects. Family 1 = Models with modulatory effects on FG→MTG, but not on FG→IFG. Family 2 = Models with modulatory effects on FG→IFG, but not on FG→MTG. Family 3 = Models with modulatory effects on both bottom-up connections. Family 4 = Models without modulatory effects on either bottom-up connection. Family 3 was the winning family (marked in bold). Second step BMS was to test families with different *top-down* modulatory effects in family 3 (the winning family). Family A = Models with modulatory effects on IFG→MTG, but not on IFG→FG. Family B = Models with modulatory effects on IFG→FG, but not on IFG→MTG. Family C = Models with modulatory effects on both top-down connections. Family D = Models without modulatory effects on either top-down connection. There was no difference between these families.

Table 6 shows the posterior family exceedance probabilities from the random effects BMS analysis in the auditory modality. Similar to the visual modality, family 3 with modulatory effects of lexical conditions on both bottom-up connections (from STG to MTG and to IFG) showed the highest evidence out of the 4 families in the first step analysis for TD (Family 3, exceedance probabilities 0.97) and RD (Family 3, exceedance probabilities 0.91). At the next step of BMS analysis, the 16 models included in family 3 were partitioned into 4 separate families (family A, B, C, D) with different modulatory effects on top-down connections. However, there was no clear evidence in favor of any family for either TD or RD.

**Table 6:** Posterior family exceedance probabilities for the typically developing (TD) and reading disability (RD) groups in the auditory modality.

		TD	RD
First step BMS	Family 1	0.01	0.04
	Family 2	0.02	0.03
	**Family 3**	**0.97**	**0.91**
	Family 4	0.01	0.02
Second step BMS	Family A	0.33	0.18
	Family B	0.22	0.16
	Family C	0.27	0.51
	Family D	0.18	0.15

Note: First step BMS was to test families with different *bottom-up* modulatory effects. Family 1 = Models with modulatory effects on STG→MTG, but not on STG→IFG. Family 2 = Models with modulatory effects on STG→IFG, but not on STG→MTG. Family 3 = Models with modulatory effects on both bottom-up connections. Family 4 = Models without modulatory effects on either bottom-up connection. Family 3 was the winning family (marked in bold). Second step BMS was to test families with different *top-down* modulatory effects in family 3 (the winning family). Family A = Models with modulatory effects on IFG→MTG, but not on IFG→STG. Family B = Models with modulatory effects on IFG→STG, but not on IFG→MTG. Family C = Models with modulatory effects on both top-down connections. Family D = Models without modulatory effects on either top-down connection. There was no difference between these families.

### Bayesian Model Averaging (BMA) and ANOVA results

Based on these above results, a BMA analysis was done for family 3 (across 16 models of families A, B, C, and D) for each subject for both modalities. All of the averaging results in this paper were obtained with an Occam's window defined using a minimal posterior odds ratio of OCC = 1/20. The Occam's window algorithm was devised primarily to allow for fast Bayesian Model Averaging. The algorithm is based on selecting a small set of subspaces from the parameter space by using posterior sampling. The posterior means of the modulatory effects from BMA estimation for each subject was entered into a next step of ANOVA analyses.

### fMRI effective connectivity differences between the TD and RD groups

Table 7 presents the posterior means of modulatory effects of the related and unrelated conditions for the typically developing (TD) and the reading disability (RD) groups in the visual modality. Table 8 presents the posterior means of the modulatory effects for the auditory modality. Modulatory effects here represent changes in the intrinsic connectivity between regions induced by the related and unrelated lexical trials. One-sample t-tests were used to examine whether each modulatory effect was significantly different from zero (p<0.05, uncorrected).

**Table 7:** The posterior means of the parameter densities on modulatory effects for the typically developing (TD) and the reading disability (RD) groups for the related and unrelated conditions in the visual modality.

TD Related				TD Unrelated			
*From:*	FG	IFG	MTG	*From:*	FG	IFG	MTG
***To:*** FG		***0.0210***	***0.0195***	***To:*** FG		0.0037	0.0023
IFG	***0.2263***		***0.0180***	IFG	***0.1232***		0.0235
MTG	***0.3258***	***0.0232***		MTG	***0.1414***	0.0163	

RD Related				RD Unrelated			
*From:*	FG	IFG	MTG	*From:*	FG	IFG	MTG
***To:*** FG		***0.0134***	0.0073	***To:*** FG		0.0014	0.0033
IFG	0.0486		**0.0239**	IFG	***0.1289***		−0.0034
MTG	0.1195	***0.0296***		MTG	***0.1113***	0.0053	

Note. FG = fusiform gyrus; IFG = inferior frontal gyrus; MTG = middle temporal gyrus. Significant effects (p<0.05, uncorrected) are marked in italic bold.

**Table 8:** The posterior means of the parameter densities on modulatory effects for the typically developing (TD) and the reading disability (RD) groups for the related and unrelated conditions in the auditory modality.

TD Related				TD Unrelated			
*From:*	IFG	MTG	STG	*From:*	IFG	MTG	STG
***To:*** IFG		***0.0242***	***0.2267***	***To:*** IFG		0.0069	***0.2695***
MTG	0.0317		***0.2938***	MTG	0.0101		***0.2177***
STG	***0.0384***	***0.0560***		STG	0.0238	0.0132	

RD Related				RD Unrelated			
*From:*	IFG	MTG	STG	*From:*	IFG	MTG	STG
***To:*** IFG		***0.0230***	***0.1657***	To: IFG		***0.0197***	***0.2058***
MTG	***0.0231***		***0.1875***	MTG	0.0096		***0.1755***
STG	0.0584	***0.0205***		STG	0.0143	0.0065	

Note. IFG = inferior frontal gyrus; MTG = middle temporal gyrus; STG = superior temporal gyrus. Significant effects (p<0.05, uncorrected) are marked in italic bold.

**Figure 1:**
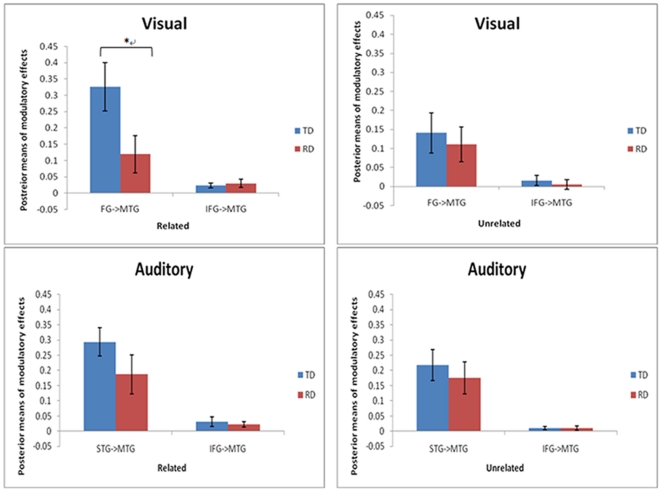
Typically developing (TD) group showed significantly stronger modulatory effects than reading disability (RD) group on the bottom-up connection from fusiform gyrus (FG) to middle temporal gyrus (MTG), but not on the top-down connection from inferior frontal gyrus (IFG) to MTG. This difference was only in the related condition in the visual modality. *, p<0.05.

We calculated 2 group (TD, RD) by 2 relatedness (related, unrelated) by 2 coupled region (top-down from inferior frontal gyrus, bottom-up from fusiform gyrus) ANOVAs for the visual modality to investigate modulatory effects to MTG. The ANOVA showed a significant group*relatedness*region (F(1, 22) = 5.530, p = .028) interaction effect, and trends toward group*relatedness (F(1,22) = 3.684, p = .068) and group*region (F(1,22) = 3.298,p = 0.083) interactions. To further understand the three-way interaction, a 2 group by 2 region ANOVA was calculated for related and unrelated conditions separately. These analyses revealed that there was significant main effect of group (F(1,22) = 4.767, p = 0.040) and significant group*region (F(1,22) = 5.630, p = 0.027) interaction effect in the related condition, but not in the unrelated condition. Follow up two-sample t-tests showed that TD group showed a significantly larger modulatory effect than the RD group in the bottom-up connection from FG to MTG (t(22) = 2.304, p = .031), but not in the top-down connection (t(22) = −0.462, p = .649) (See Fig. 1).

We calculated 2 group (TD, RD) by 2 relatedness (related, unrelated) by 2 direction (FG→IFG, IFG→FG) ANOVA to investigate the interaction between the top-down control region (IFG) and bottom-up input region (FG). This analysis revealed no group main effect or interaction effects involving group.

Using the same ANOVAs as for the visual modality, except that the input region was superior temporal gyrus, revealed no group differences or interaction effects involving group for any connections in the auditory modality.

## Discussion

Using DCM (Dynamic Causal Modeling), the present study investigated effective connectivity in the left hemisphere during semantic processing in children (8- to 14-year-olds) with reading disabilities (RD) compared to typically developing (TD) children. Children were asked to make association judgments to related and unrelated word pairs presented in the visual or auditory modality. The results revealed that children with reading disability showed weaker bottom-up modulatory effects from fusiform gyrus (FG) to middle temporal gyrus (MTG) only for related word pairs in the visual modality. There were no group differences in effective connectivity for the auditory modality.

The modality-specific bottom-up connectivity deficit with the semantic processing region in children with reading disability provides new evidence for the discrepancy between reading and listening comprehension in many of these children [1]–[4]. This finding is consistent with previous studies reporting brain abnormalities during semantic processing tasks in the visual modality [6]–[8], [42], [43], with weaker or non-significant effects in the auditory modality [9], [11]. Even though previous studies suggested functional brain abnormalities during semantic processing tasks in dyslexia in the visual modality, some studies further suggested that this semantic difficulty may not be a semantic deficit per se, but just a developmental delay due to poor word decoding skills [44], [45]. Using event-related potentials, Silva-Pereyra et al. (2003) reported that poor readers had longer reaction times and lower accuracy compared to controls during a visual word categorization task. However, there were only group differences in the P2 (reflecting attentional demands and stimulus evaluation) and P300 (reflecting stimulus evaluation and memory updating) components, but not in the N400 (reflecting semantic processing) component, suggesting that semantic processing in poor readers may not be a semantic deficit, but the late reflection of an early word decoding problem [45]. Schulz et al. (2009) reported that both the reading-level matched control group and the dyslexic group showed a similar reversal of semantic incongruency effects (sentences with incongruent endings vs sentences with congruent endings) in the inferior parietal region (in fMRI data) and similar reduced semantic incongruency effects around 400 ms (in ERP data) compared to the age-matched control group, suggesting that the semantic impairment in dyslexia resembles a developmental delay [44].

The current study also revealed that bottom-up rather than top-down connectivity deficits appear to be the core deficit in reading disability. This finding is consistent with previous neuroimaging literature suggesting that the critical deficit for reading disability is not in frontal cortex but rather in left temporo-parietal cortex. In a study that compared children with reading disability to age- and reading-matched controls, it was shown that frontal activation was only different for an age-matched comparison, but that temporo-parietal activation was different for the age- and reading-matched comparisons [22]. This suggests that differences in frontal cortex may reflect a developmental delay, whereas differences in temporo-parietal cortex may represent a developmental deviance. This finding is also consistent with two previous effective connectivity studies on phonological processing which suggested a bottom-up rather than top-down connectivity deficit in reading disability [14], [15]. Our study extended these findings by showing that bottom-up rather than top-down connectivity alteration is the core deficit in reading disability during semantic processing.

Both dual route models of reading [46] and connectionist models of reading [47] agree that access to semantic representations may be achieved either directly from orthographic representations or indirectly from orthographic to phonological to semantic representations. It has been long believed that the discrepancy between reading and listening comprehension in reading disability is due to a brain abnormality in regions involved in mapping between orthographic and phonological representations which then has a negative influence on access to semantic representations (an indirect way deficit). Whether this discrepancy may be due to deficits involved in direct mapping between orthographic and semantic representations has not been tested before. Our study suggests that the discrepancy between reading and listening comprehension in reading disability may be due in part to a deficit in the direct mapping of orthography to semantics by demonstrating reduced connectivity from fusiform cortex to middle temporal gyrus only for the related trials during visual word processing. Our task involved making judgments as to whether two sequentially presented words were associated in their meaning. We suggest that orthographic representations activate a semantic pattern for the first word. In the case of related pairs, the orthographic representation of the second word can more effectively drive the semantic system because of the overlapping semantic features in these pairs [30]. In contrast, for unrelated pairs, orthography can less effectively drive the semantic system due to the lack of overlapping features. This results a group difference in the modulatory effects for related pairs but not for unrelated pairs.

It is less likely that this group by relatedness interaction effect is due to an indirect deficit in the mapping from orthography to phonology and then to semantics in children with reading disability. If this was the case, then one would predict a group difference in modulatory effects for both the related and unrelated pairs. There is no reason to expect that the mapping from orthography to phonology would be different for related and unrelated pairs – both should be negatively affected by the decoding deficit in reading disability. Therefore, the modulatory effects to semantic representations should be equally altered in the related and unrelated pairs. However, we observed that the modulatory effects into the middle temporal gyrus, believed to be important for semantic processing, were only weaker for the related pairs in the children with reading disability. Our study does not rule out the possibility that those with reading disability have a deficit in decoding from orthography to phonology. In fact, both previous literature [6], [7], [12]–[14], [22] and the current study used decoding measures to define reading disability. Our study is unique, however, because we additionally demonstrated a deficit in reading disability in accessing meaning based representations from the direct mapping from orthography to semantics.

Our conventional analysis revealed no group differences in the intensity of activation in regions involved in orthographic and semantic processing in the left hemisphere. Our lack of differences between typically developing and reading disability children in activation in fusiform gyrus is not consistent with previous studies that have revealed differences within fusiform gyrus [6], [48]–[50]. It is possible that the conventional analysis in our study was not sensitive enough to detect activation differences in this region, so deficits in orthographic processing may have led to deficits in mapping these representations to semantics. Indeed, the reading disability children in our study showed a deficit in spelling (see Table 1), which indicates that they have orthographic processing problems.

In conclusion, this study revealed a deficit in the visual modality for children with reading disability in bottom-up connectivity from fusiform gyrus to middle temporal gyrus. Bottom-up rather than top-down connectivity deficits support previous studies suggesting that the core deficit in reading disability is in temporo-parietal cortex. This modality-specific deficit provides new evidence accounting for the discrepancy between visual and auditory word comprehension in children with reading disability. Because deficient connectivity was found only for related pairs, this suggests a possible deficit in the connection from orthography to semantics. Previous research shows that a characteristic deficit in reading disability is decoding print into sound based representations [6], [7], [51]–[53]. However, our study additionally suggests that visual word comprehension deficits in reading disability may be due in part to a deficit in the direct mapping from orthography to semantics.
